# Invasion Is a Community Affair: Clandestine Followers in the Bacterial Community Associated to Green Algae, *Caulerpa* *racemosa*, Track the Invasion Source

**DOI:** 10.1371/journal.pone.0068429

**Published:** 2013-07-16

**Authors:** Tania Aires, Ester A. Serrão, Gary Kendrick, Carlos M. Duarte, Sophie Arnaud-Haond

**Affiliations:** 1 Center for Marine Sciences, University of Algarve, Faro, Portugal; 2 School of Plant Biology, The University of Western Australia, Crawley, Australia; 3 Department of Global Change Research, Institut Mediterráni d’Estudis Avançats, Esporles, Mallorca, Spain; 4 Institut Français de Recherche pour l’Exploitation de la Mer - Technopole de Brest-Iroise, Plouzané, France; University of Melbourne, Australia

## Abstract

Biological invasions rank amongst the most deleterious components of global change inducing alterations from genes to ecosystems. The genetic characteristics of introduced pools of individuals greatly influence the capacity of introduced species to establish and expand. The recently demonstrated heritability of microbial communities associated to individual genotypes of primary producers makes them a potentially essential element of the evolution and adaptability of their hosts. Here, we characterized the bacterial communities associated to native and non-native populations of the marine green macroalga *Caulerpa* *racemosa* through pyrosequencing, and explored their potential role on the strikingly invasive trajectory of their host in the Mediterranean. The similarity of endophytic bacterial communities from the native Australian range and several Mediterranean locations confirmed the origin of invasion and revealed distinct communities associated to a second Mediterranean variety of *C* *. racemosa* long reported in the Mediterranean. Comparative analysis of these two groups demonstrated the stability of the composition of bacterial communities through the successive steps of introduction and invasion and suggested the vertical transmission of some major bacterial OTUs. Indirect inferences on the taxonomic identity and associated metabolism of bacterial lineages showed a striking consistency with sediment upheaval conditions associated to the expansion of their invasive host and to the decline of native species. These results demonstrate that bacterial communities can be an effective tracer of the origin of invasion and support their potential role in their eukaryotic host’s adaptation to new environments. They put forward the critical need to consider the 'meta-organism' encompassing both the host and associated micro-organisms, to unravel the origins, causes and mechanisms underlying biological invasions.

## Introduction

Anthropogenic disturbances inducing habitat change, modification of biotic interactions and deliberated or accidental translocation of specimens outside their species distribution range are propelling a global increase in biological invasions [1,2]. Invasive species, in turn, are additional drivers of biodiversity decline [3].

The capacity of introduced species to expand and become invasive is dependent on their capacity to adapt to new environmental conditions. Thus far, assessments of the potential of introduced species for invasive behavior have focused on the role of morphological and physiological traits as drivers of their potential to outcompete native species [4,5]. However, the competitive potential of invasive species may not be entirely determined by their intrinsic capacities, but may be at least partly shaped by associated microbes[6]. . Bacterial communities are in fact often transmitted vertically, becoming a heritable component able to greatly influence the function, competence and evolution of their host genotypes [7]. Our understanding of the mechanisms underlying the establishment and spread of introduced species may therefore require a serious appraisal of the potential co-introduction and influence of bacterial communities on the success of non-indigenous species.

Alga from Caulerpa genus are showing a complex morphology consisting of - leave like structures-fronds, stolon and rhizoids, yet with the entire thallus composed of a single cell, a giant siphonous structure that was previously showed in association with an important bacterial diversity on the invasive alga *Caulerpa* *taxifolia* [8]. In this pioneer study similarity in the composition of the highly diverse bacterial communities in native populations from Northern tropical Australia and those introduced in the Mediterranean [8] was reported, in line with the genetic similarity of the host algae from these two regions [8,9,10]. The methods used did not allow the exhaustive characterization of bacterial diversity, or the discrimination of endophytic *versus* epiphytic or host *versus* habitat specific bacteria, but suggested that bacterial community could be used to trace back the origin of introductions. Invasive species frequently act as ecosystem engineers [11] and the presence of *Caulerpa* species induces sediment modifications that are suspected to increase their success and contribute to the displacement of native species [5,6]. Among others, *Caulerpa* species enhance sulphate reduction rates and the production of sulfide, rendering the sediment highly toxic to seagrasses [12,13].

Epiphytic bacteria on algae are variable and distinct from those in seawater [14,15,16] and play key roles on in morphological development [17,18,19,20], growth and nutrient acquisition [21,22] spore release and settlement [23,24], and protection from fouling [25], among others. Contrastingly and although they are more likely to present tight association to their host and possible vertical transmission, progresses in characterizing endophytic microbial communities associated to photosynthetic organisms, including macrophytes, have been hindered by the difficulties associated with strain cultivation and high chloroplast contamination in endophytic bacterial libraries [26]. These technical challenges hitherto impeded the efficient use of Next Generation Sequencing for massive 16S characterization. A recent solution to this technical challenge [27] now allows the comprehensive characterization of bacterial communities associated with *C* *. racemosa* in the Mediterranean Sea and in one of the native ranges of the variety *C* *. racemosa* var. *cylindracea* , endemic from the southern and northern coasts of Western Australia, northeastern Australia and New Caledonia [28,29]. We identified and compared putative bacterial epiphytic and endophytic communities in the native and invaded ranges, in order to test for the Australian origin of the invasive Mediterranean variety and the stability of the associations between host lineages and endophytic bacterial communities, and to identify possible strains that may have passively or actively participated in the invasive trajectory of their host.

## Results

**Figure 1:**
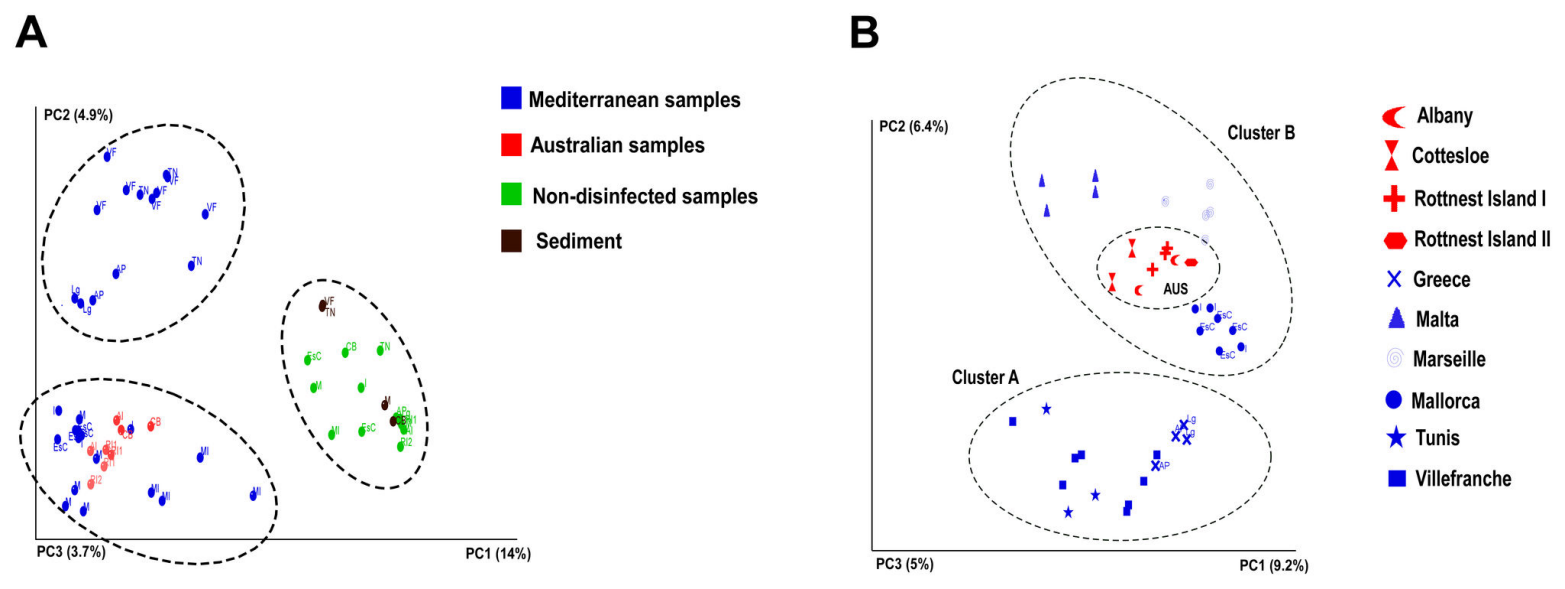
PCA representing weighted Unifrac analysis of *C. racemosa* samples from Mediterranean Sea and Australia (invaded and native range), A-All samples including controls; B-Disinfected samples only. Sample Codes: **Al**- Albany (Australia), **CB**- Cottesloe Beach (Australia), **Lg**- Liguria (Greece), **AP**- Agios Pavlo (Greece), **Ml**- Malta, M-Marseille, **I**- Illetas (Mallorca, Spain), **EsC**- Es Cargol (Mallorca, Spain), **RI1**- Rottnest Island 1 (Australia), **RI2**- Rottnest Island 2 (Australia), **TN**- Tunisia, **VF**- Villefranche (France).

**Figure 2:**
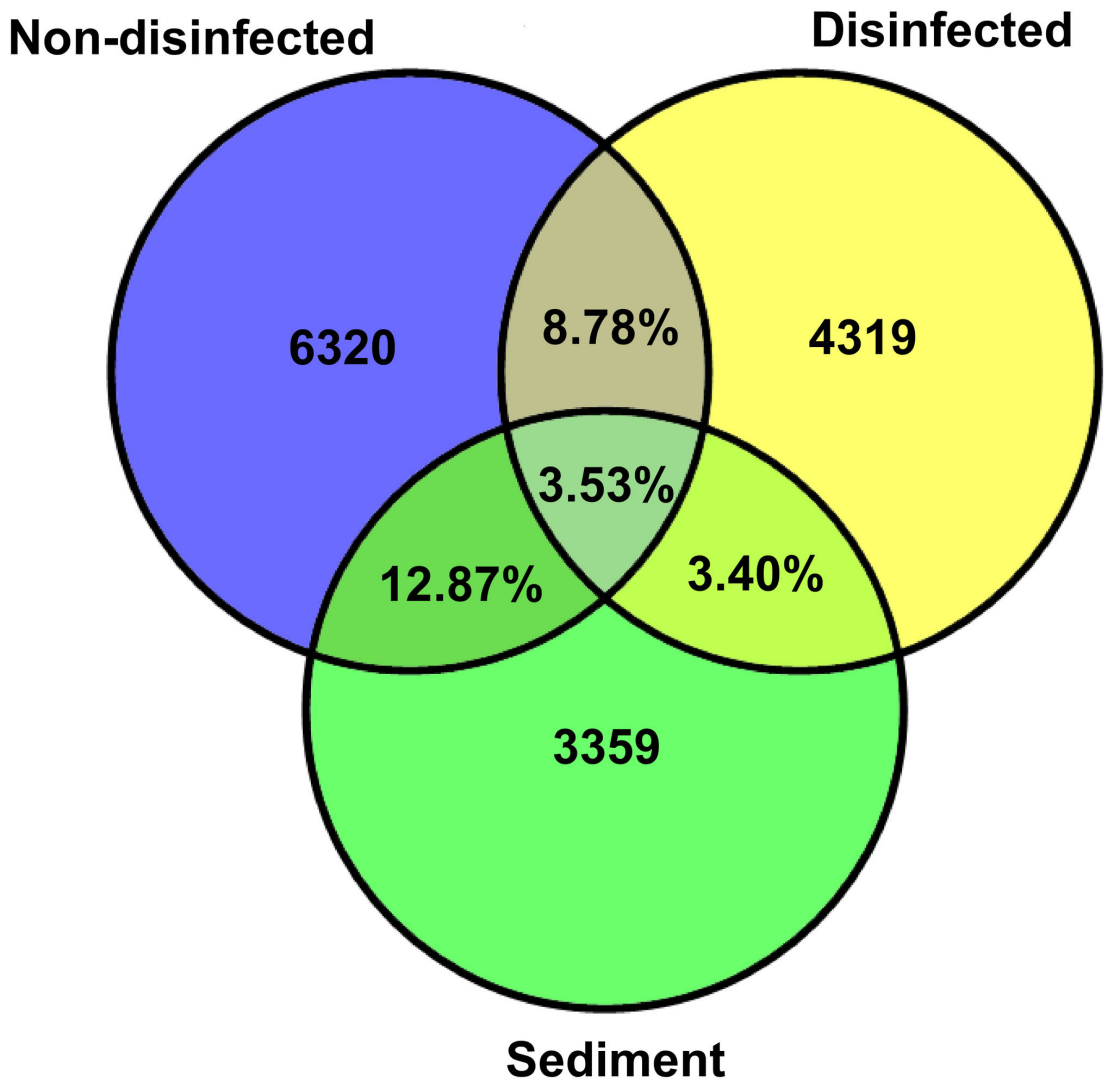
Venn diagram representing bacterial communities shared within the three different treatments. Numbers on each treatment represent the number of OTUs and percentages on overlapping areas represent the percentage of shared OTUs.

The total of 173512 sequences used in downstream analysis after quality control (Table S1) revealed 18325 bacterial Operational Taxonomic Units (OTUs) (represented by unique 16S genotypes) that segregate into three distinct clusters (Figure 1A). These results allowed us to distinguish total communities including epiphytic bacteria (i.e., samples from non-treated algae) and sediment samples *versus* the other two groups composed of endophytic communities (i.e., bleached algal samples free of epiphytes and of chloroplasts). In order to understand how endophytic bacteria would shape the different populations, the same analyses were applied just on the disinfected samples allowing us to look deeper into the endophytic community structure. Results show that populations were separated into 2 clusters. Cluster A gathered samples just from Mediterranean sites including Tunis, Villefranche and Greece, and Cluster B joined samples from Malta, Marseille and Mallorca with samples from Australian native range (WA) (Figure 1B). This discrimination of three groups by the Principal Component Analysis (PCA) was strongly supported (p<0.01) by the analysis of community similarity (ANOSIM) using Bray-Curtis distances (Table S3). The clustering of non-disinfected and sediment samples apart from disinfected samples suggests the distinct composition of endophytic compared to putative epiphytic communities that clearly appear to be more similar to the environmental ones (from sediment) (Figure 1A). The Venn diagram drawn from an OTU table pooling samples from the different treatments, shows that non-disinfected samples and sediment share the highest percentage of OTUs -12.87% (Figure 2). In the case of the percentage of OTUs shared between disinfected and non-disinfected samples (8.78%) (Figure 2) it is mostly driven by OTUs that appeared as shared due to a single sequence in the first pool *vs* thousands of the same OTU in the other, reflecting the very scarce persistence in the surface disinfected samples of some bacteria that are mostly epiphytic.

**Figure 3:**
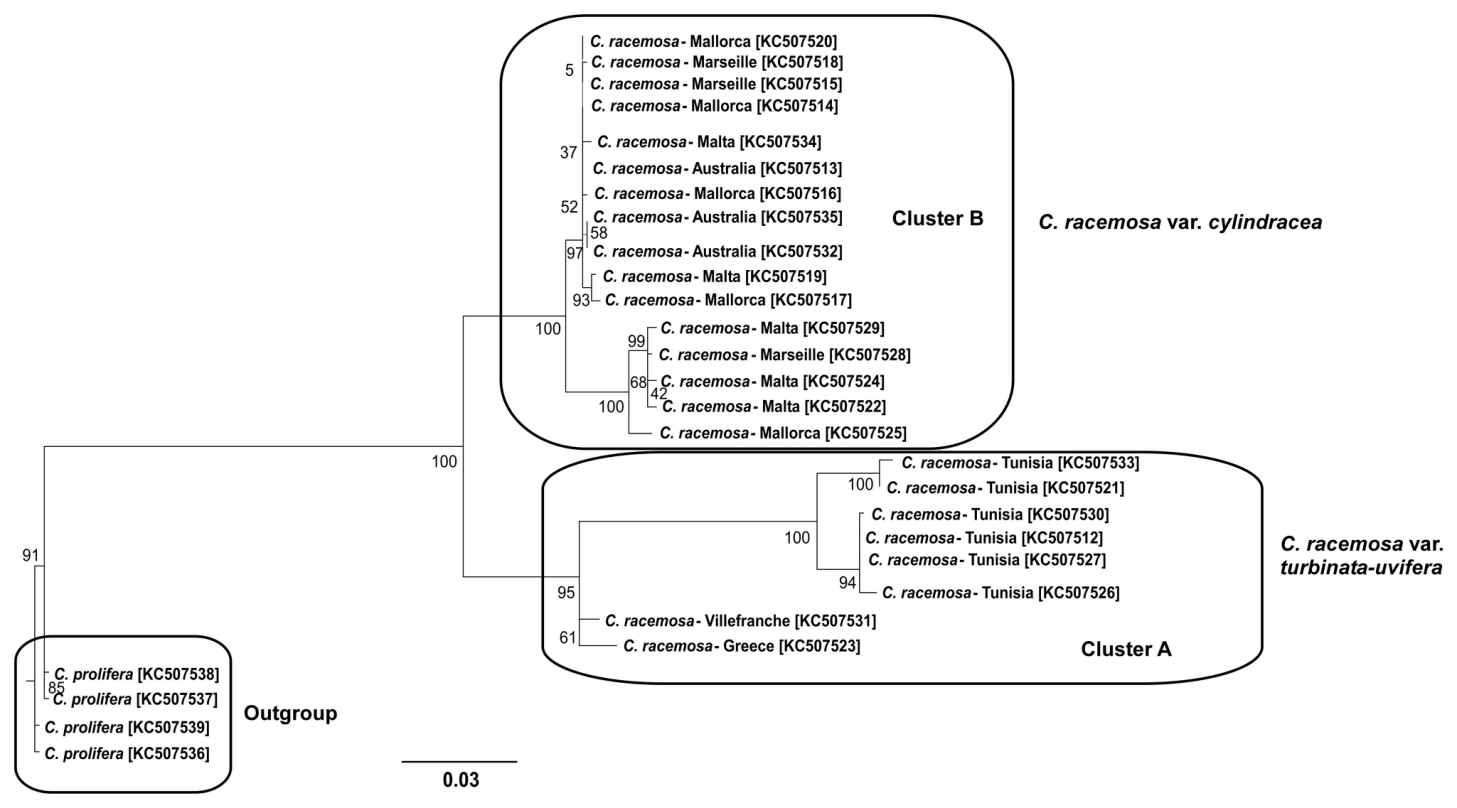
Maximum Likelihood tree from *C. racemosa* ITS calculated using the evolution Model TPM2+G with bootstraps calculated after 1000 replicates. Alignments of each cluster were BLAST against Genebank nucleotide database and got the highest hits with sequences from Verlaque et al. 2003 study [AY334305-Cluster A and AY173118-Cluster B] *C. prolifera* samples from Mallorca were used as outgroup. Genbank accession numbers are represented within brackets.

Some of the sampling units from the different locations were amplified for 18S rDNA and sequences were compared to the Genbank database in order to identify the phylogenetic identity of SUs from the different collection sites. Interestingly, the same dichotomy found for the endophytic bacterial communities (Figure 1B) was reflected by the host phylogeny, where two different clusters separating samples from Tunis, Villefranche and Greece and samples from Malta, Marseille and Mallorca and those from the Australian native range (Figure 3). SUs gathered in phylogenetic Cluster A were identified as being phylogenetically related to *C* *. racemosa* var. *turbinata* *-uvifera* while sequences in Cluster B were identified as being related to *C* *. racemosa* var. *cylindracea* (Figure 3) which is the variety described to be native from Western Australia. This phylogenetic approach, besides allowing us to compare the population structure with that from endophytic bacteria, also allowed us to correctly identify the different *C* *. racemosa* varieties that were misidentified by the collectors.

**Figure 4:**
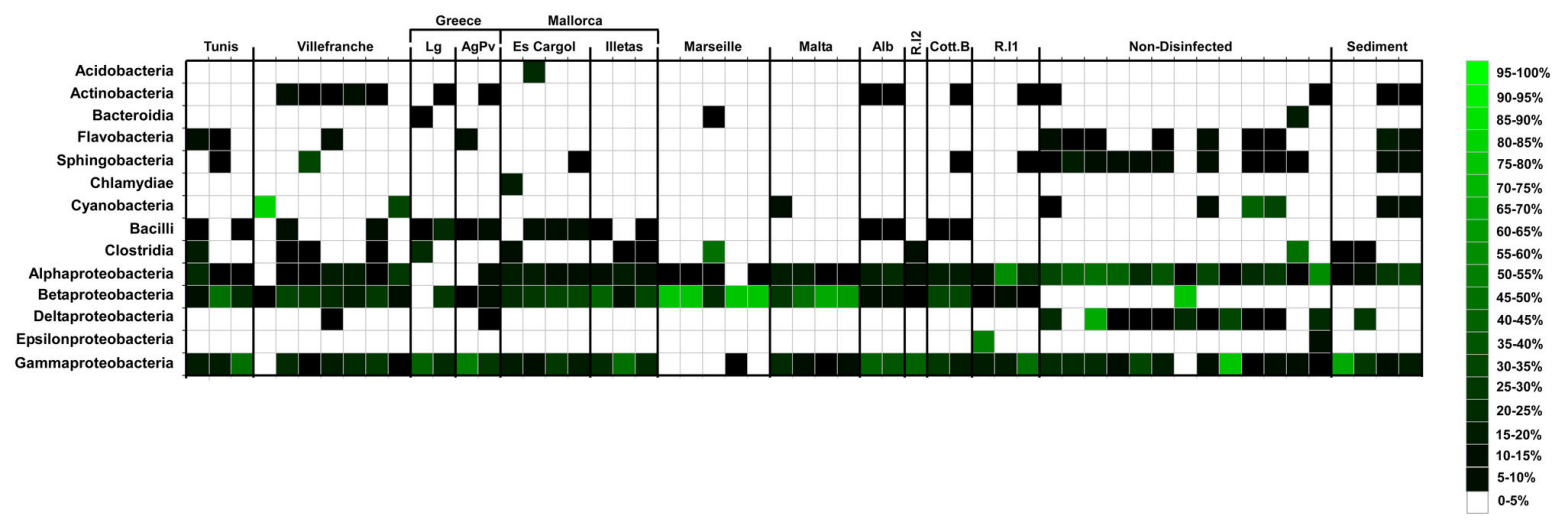
Heatmap representing distribution of the main classes among samples. Scale bar represents the percentage of sequences belonging to the OTUs represented in the heatmap.

Heatmaps built for both the most common classes and orders (Figure 4 and Figure S1) show that bacterial communities, at these taxonomic levels, mainly distinguish disinfected and non-disinfected samples. At the class level, Flavobacteria, Sphigobacteria and Deltaproteobacteria were clearly prevalent in non-disinfected samples while almost absent in disinfected SUs (Figure 4). The Heatmap for the order level show that Flavobacteriales, Sphingobacteriales and Rhodobacterales were the most prevalent orders for non-disinfected samples with just a few records on endophytic community (Figure S1). Betaproteobacteria was one of the most ubiquitous classes found within the bacterial community of disinfected SUs (Figure 4), mostly due to the order Burkholderiales, common to samples from all sampling sites but one (Rottnest Island 1) while nearly absent from non-disinfected ones (Figure S1).

Only at the order level it was possible to find a pattern that resembles that given by the whole bacterial community (the Clusters A and B). The order Pseudomonadales was conspicuously more present in *C* *. racemosa* from Tunis, Villefranche and Greece (Cluster A) and the order Vibrionales was found only in samples from the previously determined Cluster B, except for *C* *. racemosa* sampled in Marseille and one of the locations in Mallorca (Figure S1). Interestingly, phylogenetic results for some of the most ubiquitous lineages belonging to *Burkholderiales* (Figure S2) were segregated between the two varieties of their algal host with the two clusters (A and B) identified and associated either to the invasive Australian or to the ancient Mediterranean variety (Figure S2), supporting a possible co-evolution of host and bacterial lineages.

Within Burkholderiales order the most represented family was Comamonadaceae which phylogenetic tree showed a cluster exclusive of the invasive group B, while the other showed a more complex distribution among groups (data not shown). The Comamonadaceae include nitrogen-fixing and nitrate-reducing bacteria [30]. Within this family, the most ubiquitous OTU (OTU110, 230 base pair) had highest similarity (99%) with *Alicycliphilus* *denitrificans* (GenBank CP002657.1; on the whole 230 bp fragment). A network of OTU110 sequences revealed three clusters (Figure 4), two almost exclusively composed by sequences from the ‘invasive’ variety (cluster B), while a third one was, with one exception, entirely composed of sequences from the old Mediterranean variety (cluster A) from Tunis, Villefranche or Greece. No sequences were common to both varieties; the only nodes of the network shared between varieties were due to the collapse of similar sequences during the star-contraction procedures. Contrastingly, haplotypes found in Australian and Mediterranean *C* *. racemosa* var. *cylindracea* belonged to the same two remaining clusters and a large amount of them were shared.

## Discussion

Results reported here show an extreme diversity of bacterial OTUs associated to *C* *. racemosa* (Table S2) both in its native and in its invasive range, reaching or exceeding the already high level of diversity reported for exceptional holobionts such as coral or sponges systems [24]. Also, the diversity of the bacterial community found in this study, overcomes the already large diversity found for another siphonous alga – *Bryopsis* – [31,32,33]. However, the use of DGGE in this study like for the majority of studies aiming to characterize bacterial endophytes thus far may have led to underestimation of the bacterial diversity. This is usually more thoroughly revealed by NGS approaches, possibly mainly due to the difficulty to resolve overlapping bands in DGGE [34]. The impressive level of bacterial diversity is still observed even when considering only endophytic communities and not the putative epiphytic ones forming the algal biofilm that here clusters with sediment communities (Figure 1A and Figure 2).

The differentiation of endophytic communities among varieties suggests that their composition is strongly influenced by the nature of the host, with significant geographical segregation also marginally revealed within groups (Table S4), indicating either divergence or selection of some symbiont lineages, or the influence of some environmentally acquired strains in the community (Figure 1B). Several recent studies demonstrated that, when epiphytic bacteria are isolated from the host and compared among different species, host phylogeny is more related to the composition of epiphytic community than to the region of origin [14,15,32,35,36]. This appears also true when bacteria composition changes seasonally [16,36].

Our results therefore indicate algae can also be considered as a reservoir of bacterial diversity, in tight association to their hosts for endophytic ones which together with the similarity of epiphytic and sediment communities, echoes the recent findings on the siphonous green macroalga *Bryopsis* [31]. Besides, the strong clustering of communities according to varieties and treatments is supported here by a high statistical support thanks to the use of multiple replicates of high throughput community characterization for both varieties in each sampling location (Table S1, S3 and S4).

**Figure 5:**
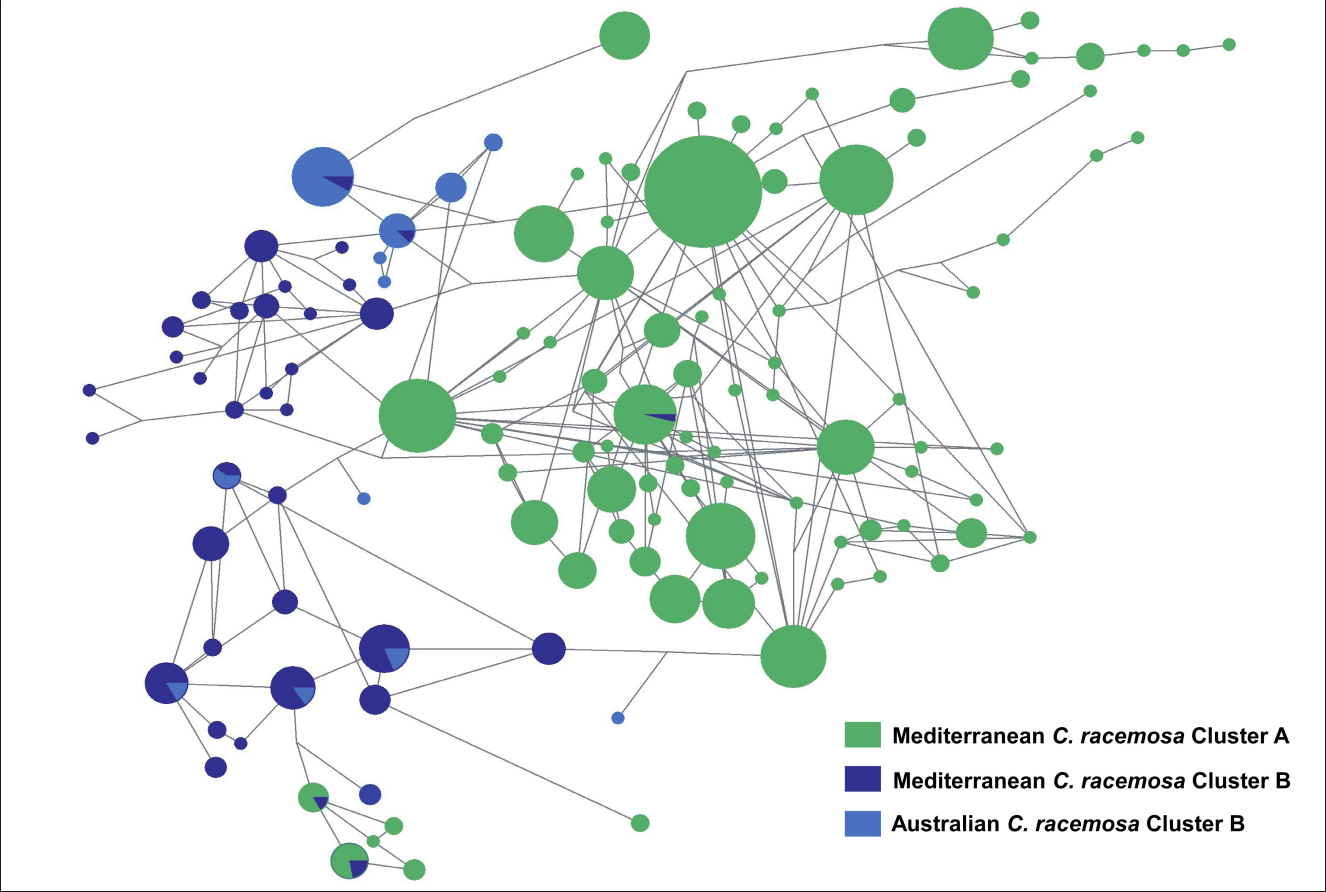
Network of haplotypes from the most ubiquitous OTU (110) in the set of OTUs represented in *Comamonadaceae* family phylogenetic tree. Network was drawn without keeping distance of links proportional to the number of mutations, in order to illustrate the clustering rather than the divergence

The origin and vector for the invasive variety of *Caulerpa* *racemosa* has been, for a long time, an open question, with the Red Sea or Australia as potential origins [28] and ballast water, aquarium trade, or Lessepsian migration as possible vectors and pathways. The central position in group B of Australian communities, associated to the ‘invasive variety’ *C* *. racemosa* *var.* *cylindracea* from the Western Mediterranean (Figures 1B, 3 and S2), confirms the most recent phylogenetic evidence supporting its western Australian origin [28]. Group A, encompassing communities characterized in other Mediterranean locations (Figures 1B, 3 and S2), is significantly distinct and associated to the variety recognized as *C* *. racemosa* var. *turbinata* *-uvifera*, an ‘ancient Mediterranean’ variety of unknown origin, first described in the early XX^th^ century in Tunis [28]. Besides this significant differentiation in two clusters showing different community composition, the bacterial community associated to the ‘invasive variety’ *C* *. racemosa* *var.* *cylindracea* from the Western Mediterranean indeed shared a higher percentage of the OTUS with the Australian ones than with the communities associated to the other Mediterranean variety, providing additional evidence of their common origin (Figure 1B Table S4). In addition to the clustering of communities as a whole, the phylogenetic analysis of several of the most ubiquitous lineages of OTUs observed in these communities also confirmed the dichotomy between the communities associated to each variety of *C* *. racemosa* (Figures 5 and S2). Finally the segregation of haplotypes within some of these OTUs also showed a striking congruence with their host lineage, also in line with the hypothesis of co-evolution of host and some bacterial lineages (Figures 5 and S2).

Besides possible transmission of some of the endophytes during sexual events that cannot be inferred from results reported here, vertical transmission is likely to be favored by the partially clonal reproduction regime of *C* *. racemosa* through clonal fragmentation. Clonality is a life history trait recognized as a potential facilitator of invasion through enhanced spreading capacity after founder events [4] it may be suggested here that such mating system may also facilitate the transmission of bacterial communities.

Bacterial characterization by 16S amplification does not always yield the most detailed information about the strains associated and the lower taxonomic levels are usually missed [37], which sometimes makes the comparison between bacteria associated to different hosts only possible at higher taxonomic levels. We compared dominant lineages with those characterized on *Bryopsis* [31], which is also a green coenocytic algal genus. They found three main bacterial lineages to be intimately associated to *Bryopsis* species where endophytic communities were dominated by strains belonging to Bacteroidetes, Flavobacteriaceae and Xanthomonadaceae [31,32]. Contrastingly in the present study the two latter groups are associated to non-disinfected samples, supporting those as surface associated bacteria, in agreement with previous findings on other algal species (e.g. *Fucus vesiculosus*, *Gracilaria* *vermiculophylla* and *Ulva* *intestinalis* ) [36,38,39]. Stratil et al (2013) [16] found the relative abundance of Rhodobacteraceae strains of epibacterial community to be positively correlated to temperature changes in *Fucus vesiculosus*, a brown alga. Our results show the presence of strains belonging to Rhodobacterales order in almost all non-disinfected samples (all having been collected during summer periods) (Figure S1) which raises the hypothesis that the importance of this strain in the biofilm during warmer periods might be generalizable to some green algae in addition to the brown lineage. A high percentage of Alphaproteobacteria (Figure 4) and Bacteroides (Sphigobacteriales and Flavobacteriales; Figure S1), similar to our epiphytic communities, associated to *F* *. vesiculosus* during summer [36]. Interestingly, a negligible presence of Betaproteobacteria was detected in our non-disinfected samples, similarly to on *Fucus vesiculosus* [16], these were the most conspicuous OTUs among our endophytic bacterial communities. These findings suggest a endophytic specificity of this bacterial class [40], a hypothesis that deserves further investigation requiring analysis of endo versus epiphytic bacterial community on this and other brown algae.

Bacterial metabolism cannot be inferred from 16S characterization only, although it is difficult to ignore here that some OTUs including the most dominant and ubiquitous ones show high similarity (>99%) with bacterial strains characterized for metabolic functions strikingly fitting the critical alterations of sediment associated to the presence of *Caulerpa sp.*, such as nitrate reduction in anaerobic conditions [12,41] or sulfate reduction. The invasiveness of *Caulerpa* species in the Mediterranean involves the competitive displacement of seagrasses, prominently *Posidonia* *oceanica* [5,6]. The OTU110, for example, showed a high similarity (>99%) with *Alicycliphilus* *denitrificans* , a nitrate-reducing betaproteobacterium able to perform in anaerobic conditions similar to those described in sediment colonized by *Caulerpa* sp. [12]. Moreover, the endophytic flora of *Caulerpa* *racemosa* also includes a variety of other functionally relevant bacteria, including N_2_-fixing bacteria. Additionally, seagrasses in general, and *Posidonia* *oceanica* in particular, are extremely sensitive to sulfide, which is toxic to these plants [13,42,43]. A number of OTUs identified in the endophytic flora of *Caulerpa* *racemosa* are associated with sulfur cycling. Endophytic communities exhibited a limited but ubiquitous presence of OTUs assigned to the *Desulfobacteraceae*, that commonly reduce sulfate to sulfide, with a specific cluster common to some Australian and Mediterranean samples from the invasive group B. A high diversity of OTUs assigned to *Desulfobacterales* was additionally observed in non-disinfected samples and may also be part of the biofilm associated with algae, known to contribute to important metabolic processes [35]. Indeed, *Caulerpa* species have been reported to enhance sulphate reduction rates in the sediments they colonize, rendering them unsuitable for sulfide-sensitive *Posidonia* *oceanica* [12]. The results presented here suggest that the capacity of *Caulerpa* to competitively exclude *Posidonia* *oceanica* by modifying the sediments may be due to endophytic bacteria.

This study reveals a large diversity of bacterial communities associated to the invasive *C* *. racemosa* , with a striking differentiation of putative epiphytic communities forming the biofilm and apparently largely similar to communities found in the sediment compared to endophytic communities which profile at all levels, from the whole community to the OTU one, indicate tight association to their eukaryotic host. This demonstrates the stability of bacterial communities during the course of transport and invasion, also indicating their potential to trace the origin of invasion. These results add to recent evidence that the bacteria flora of macro-organisms, including human, is unique, even at the level of individual, and provides an important potential as a tool in identification of taxonomic entities and pathways of migration [15,32,33,36,44].

These results also reveal some dominant and ubiquitous bacterial strains that, although their metabolism cannot be strictly inferred from 16S similarity alone, show striking coherence with the reported impacts of *Caulerpa* colonization on sediment biogeochemistry [12,45], eventually displacing seagrass [12]. Also, a majority of strains, found in this study, lying in the Betaproteobacteria class encompassing a large number of known plant-symbiotic bacteria as legume-nodulation bacteria in general and *Burkholderia* in particular [40,46] may indicate the hypothesis of those to act as enhancers of the invasive potential. This order includes common endosymbionts classically associated to plants and algae [47,48,49] and known to include nitrogen-fixing and plant/algae growth enhancer endosymbionts [50].

Recent phylogenetic studies on the *C* *. racemosa* *-peltata* complex to which this invasive variety belongs, revealed that native range of *C* *. racemosa* var. *cylindracea* is much wider than thought, with its presence confirmed in northeastern Australia and New Caledonia [29] as well as a new possible invaded area in Port Adelaide, South Australia [29]. In light of these very new findings, we suggest that a more complete sampling including these newly identified native and invaded ranges would add further important information to more precisely identify the origin of Mediterranean invasion. However, our results, allied to other recent studies [32,33], suggest that our understanding of biological invasions need to evolve from a focus on the competitive capacities of the invasive species, to consideration of the ‘meta-organism’, or ‘holobiont’ including the synergies between the host and associated bacterial communities determining their adaptation to their new environment and their capacity to outcompete native organisms.

## Materials and Methods

### Sampling and samples preparation

No specific permissions were required for sampling *Caulerpa* *racemosa* in the Mediterranean Sea locations, where this species is considered an invasive and, as such, doesn't have any law associated to its harvesting. Caulerpa racemosa is native from Western Australia however is not considered nor an endangered or protected species. A permit was provided to Dr. Gary Kendrick, from University of Western Australia, by the Department of Environment and Conservation (DEC) for a license to take or disturb flora for scientific purposes in all the locations where the sampling was carried out (Albany, Perth and Rottnest Island).

A total of 38 sampling units (SUs, set of interconnected fronds, rhizoids and stolons, also called ‘ramets’) of *Caulerpa* *racemosa* identified by the collectors as var. *Cylindracea* were analyzed from 6 locations in the Mediterranean Sea and 3 locations in Southwestern Australia (Tables S1 and S2). Sampling for each SU was done leaving at least a distance of 1m from each other. Each SU included several ramets with all the morphological parts of the algae and was kept isolated in individual zip-lock bags when sampled. Bags were then stored at -80ºC until processing. Sediment samples were also analyzed to be used as environmental control. Following a pilot study indicating a tighter association of endophytic bacteria to the host and a higher similarity of putative epiphytic ones to environmental (sediment) samples, algal material was disinfected adapting a “bleach protocol” [27,51] prior to extraction, allowing discarding both epiphytic communities and chloroplastidial DNA to concentrate on endophytic communities. In order to still include a control for the exclusion of epiphytic communities in the present study some of the SUs were split in two complete fragments (each including one or several complete ramets with all morphological part of the algae), one was treated as detailed here above for the characterization of endophytic communities while another set of ramets was not disinfected and used as a control. Bacterial DNA extraction was performed using FastDNA^®^ SPIN Kit for Soil (MP biomedicals LLC). Detailed Methodology for sampling strategy and sample preparation is provided in Material and Methods S1.

### Next Generation sequencing and Metagenomics analysis

Extracted DNA was submitted to Biocant (Cantanhede, Portugal) to be analyzed through tag-Pyrosequencing (GS FLX Titanium, 454-Life Sciences-Roche technology^®^) after amplification with modified primers for region V4 of 16S rRNA [52]. PCR amplification of the hypervariable V4 region of the 16S rRNA gene was performed using the 8bp key-tagged. After sequences’ quality control and chimera removal by Chimera Slayer, all analyses were performed using the program QIIME: Quantitative Insights Into Microbial Ecology [53]. Sampling diversity and specific richness was assessed by calculating Chao1 and Shannon indexes. To assign each OTU to the closest matching described ‘species’, BLASTN searches were performed against SILVA database and sequences were putatively assigned to a described taxa provided their blast was associated to a minimum e-value threshold of 0.001 (default value). Beta-diversity was also calculated on Qiime using the weighted Unifrac algorithm which uses qualitative measures the phylogenetic distance between sets of taxa in a phylogenetic tree [54] and PCA 2D plots were constructed to visualize data. Statistical differences between OTUs hits of different replicates were assessed by One-way ANOSIM performed using PAST (Ver. 2.16) [55]. 16S sequences from the most common order and family to all disinfected samples (Burkholderiales and Comamonadaceae respectively) were used to build phylogenetic trees with Qiime’s script make_phylogeny.py and using, by default, FastTree [56] and root was chose by the tree method default from Qiime. An OTU table was constructed by pooling the samples from different treatments (disinfected, non-disinfected and sediment) and a Venn diagram was constructed using Venny [57] in order to assess percentage of OTUs shared between different treatments. Metadata was submitted to The European Nucleotide Archive (ENA) in the Sequence Read Archive (SRA) and is available under the following accession number: ERP002264 [http://www.ebi.ac.uk/ena/data/view/ERP002264].

### Phylogenetic analysis of *Caulerpa* *racemosa* varieties

A 1100 bp amplified region containing the 3’ end of the 18S rDNA, including the intron (100 to 108 bases), the ITS1 (112 to 136 bases), 5.8S rDNA, ITS2 (281 to 315 bases), and the 5’ end of the 28S rDNA from *C* *. racemosa* , was amplified according to the PCR conditions by Verlaque et al. 2003 and cloned using pGEM-T Easy. Sequences were aligned using CLUSTAL X [58] and Maximum Likelihood analysis performed on PHYML ver. 3.0 [59] after evolution model selected using MODELTEST [60]. Alignments of each cluster were BLAST against Genbank nucleotide database in order to identify the most probable variety. Sequences were made available on GenBank database with the accession numbers KC507512 – KC5077539.

### Network

The whole set of sequences corresponding to highly represented OTUs were extracted in order to build a haplotype network and screen the nature and phylogenetic relationships of that ubiquitous family of OTUs, which are also most influent the differentiation of both groups of communities, in order to discriminate the lineages tightly associated to hosts rather than habitat dependent, and to provide a first step towards the identification of putative symbiotic lineages on the basis of their blast identification and consequent taxonomic assignment. The choice of a network of haplotype was driven by the short length of sequences obtained through pyrosequencing (usually <250 bp), that do not allow robust phylogenetic reconstruction, and by the question to be addressed. Rather than detailing the evolutionary history of lineages we indeed aimed at testing for identity and/or clustering of bacterial lineages between native and Mediterranean invasive specimens of *Caulerpa* *racemosa* var. *cylindracea* compared to those belonging to *C* *. racemosa* var. *turbinata* *-uvifera*. Alignments were processed using MUSCLE [61] alignment in Geneious Pro v 5.4 [62]. Highly divergent sequences after alignment (>3%) were excluded. Identical sequences were clustered in DNA Sp [63] and exported to Roehl format in order to build a network of haplotypes using the software Network [64]. The preprocessing option implemented in Network was used for Star contraction [64], using a radius maximum size of 5. Due the frequent instances of multiple possible characters for numerous sites (i.e. not binary), the median joining procedure was implemented and followed by a MP procedure to remove the unnecessary median vectors and links [65] and reduce the complexity of the network to improve its visualization.

## Supporting Information

Figure S1 Heatmap representing distribution of the main orders among samples. Scale bar represents the percentage of sequences belonging to the OTUs represented in the heatmap.
(TIF)

Figure S2 (TIF)

Material and Methods S1 Detailed Methodologies. (DOCX)

Table S1 Number of sample units (SUs) processed per locality. Placehold legend. Remove.
(DOCX)

Table S2 Results of samples’ quality filtering and α-diversity analyses. Placehold legend. Remove.
(DOCX)

Table S3 Statistical results of One-way ANOSIM with Bray-Curtis distance measures applied to each group of treatments’ replicates, using OTU hits. Analysis made with 9999 permutations.
(DOCX)

Table S4 Statistical results of One-way ANOSIM with Bray-Curtis distance measures applied to each group of sites replicates (only considering disinfected samples), using OTU hits. Analysis made with 9999 permutations.
(DOCX)
