# Supplementary figures and images for: Invasion Is a Community Affair: Clandestine Followers in the Bacterial Community Associated to Green Algae, Caulerpa racemosa, Track the Invasion Source
Source: PLoS One. 2013 Jul 16;8(7):e68429. doi: 10.1371/journal.pone.0068429 (PMC3713043; doi:10.1371/journal.pone.0068429)

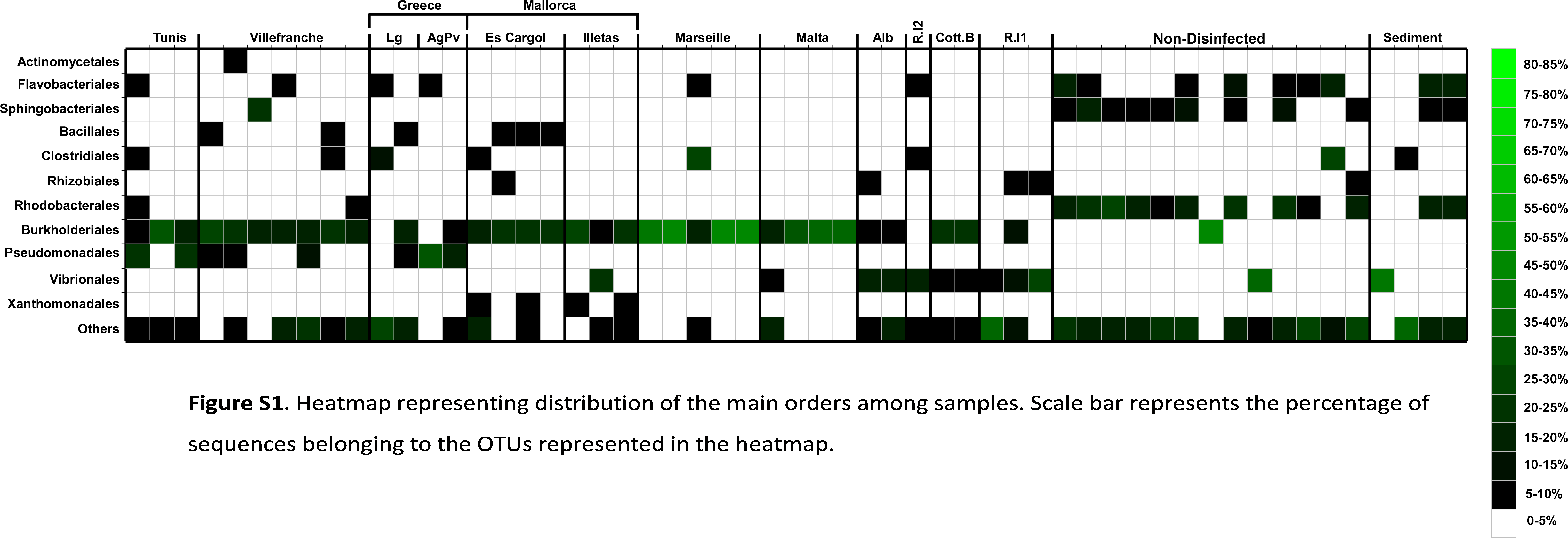

Supplement: Figure S1 — Scale bar represents the percentage of sequences belonging to the OTUs represented in the heatmap. (TIF) [file pone.0068429.s001.tif]
