# Supplementary material for: Invasion Is a Community Affair: Clandestine Followers in the Bacterial Community Associated to Green Algae, Caulerpa racemosa, Track the Invasion Source
Source: PLoS One. 2013 Jul 16;8(7):e68429. doi: 10.1371/journal.pone.0068429 (PMC3713043; doi:10.1371/journal.pone.0068429)
Supplement: Material and Methods S1 — (DOCX) [file pone.0068429.s003.docx]

**Material & Methods S1 [Detailed Methodologies]**

Sampling was performed during the summer of 2009 for the Mediterranean samples (July/August) and the summer of 2010 for the Australian samples (January/February). All the Mediterranean sampling was done by scuba diving while Australian sampling was done in intertidal zones or by snorkeling when algae were in the subtidal zone. Due to the possibility of clonal reproduction, specimens of both species were sampled with, at least, 1 meter distance to decrease the probability of sampling the same individual. Sampling sites are discriminated in Table S1.

Samples were kept refrigerated while of field and stored at -80ºC until DNA extraction.

Each sampling unit (SU) was cleaned and disinfected in order to remove epiphytic bacteria using a ‘‘bleach-ethanol pretreatment’’ developed by Coombs and Franco (2003) and readjusted by Aires et al. (2012) prior to DNA extraction. This consisted of three simple steps with modifications developed to avoid damaging the algal material: (i) the material was placed in a bath of 99% ethanol during 1 min, (ii) the sample was subsequently transferred for 5 min to a 3% bleach (diluted in seawater) solution, and (iii) the sample was immersed for 30 s in a 99% ethanol solution and rinsed with sterile seawater. To evaluate the efficiency of this method in removing epiphytic (i.e., external) bacteria, samples were stained for 5 min with 0.1 mg.mL^-1^ 4’6-diamidino-2-phenylindole (DAPI) and subsequently viewed under a confocal and fluorescence microscope with an ApoTome.2 (Zeiss, Germany) to determine whether or not the outer surface bacteria were effectively eliminated by the sterilization protocol applied. Differential interference contrast microscopy (DIC) (Axiovert 200 MAT Microscope; Zeiss, Ontario, NY, USA) was also performed.

Samples used as a control for the sterilization process were also cleaned for other algae, sediment and other macroorganisms that could be attached with the help of a tooth brush.

Sediment samples were got in four of the sampling sites (Table S2) and collected around the samples making sure that no macroorganism or part of it was mixed in the sediment.

Bacterial DNA from all the samples (including sediment) was extracted using FastDNA^®^ SPIN Kit for Soil (MP Biomedicals LLC) following the product instructions.
