# Supplementary material for: Invasion Is a Community Affair: Clandestine Followers in the Bacterial Community Associated to Green Algae, Caulerpa racemosa, Track the Invasion Source
Source: PLoS One. 2013 Jul 16;8(7):e68429. doi: 10.1371/journal.pone.0068429 (PMC3713043; doi:10.1371/journal.pone.0068429)
Supplement: Table S1 — Placehold legend. Remove. (DOCX) [file pone.0068429.s004.docx]

**Table S1-** Number of sample units (SUs) processed per locality

| **Sampling locality** | **Smaller scale locations** | **Nº of samples sequenced** |
| --- | --- | --- |
| Crete | [Liguria+Agios Pavlo] | 2+2 disinfected/1+1 non-disinfected |
| Mallorca | [Es Cargol+Illetas] | 4+2 disinfected/2+1 non-disinfected |
| Villefranche | [GPS :43° 41' 800 N and 7° 19’ 338 E and GPS : 43° 41’ 656 N and 7° 18’ 560 E] | 7disinfected |
| Marseille | [Marseille Biological Station, Le Veyron] | 2+2 disinfected/1+1 non-disinfected |
| Tunis | Sidi Daoud | 3 disinfected/1 non-disinfected |
| Malta | Anchor Bay | 4 disinfected/1 non-disinfected |
| Albany | Gull Island | 2 disinfected/1 non-disinfected |
| Perth | Cottesloe Beach | 2 disinfected/1 non-disinfected |
| Rottnest Island | Beach1 | 3 disinfected/1 non-disinfected |
| Rottnest Island | Beach2 | 1 disinfected/1 non-disinfected |
