# Supplementary material for: Invasion Is a Community Affair: Clandestine Followers in the Bacterial Community Associated to Green Algae, Caulerpa racemosa, Track the Invasion Source
Source: PLoS One. 2013 Jul 16;8(7):e68429. doi: 10.1371/journal.pone.0068429 (PMC3713043; doi:10.1371/journal.pone.0068429)
Supplement: Table S2 — Placehold legend. Remove. (DOCX) [file pone.0068429.s005.docx]

**Table S2-** Results of samples’ quality filtering and α-diversity analyses

| **Sample code** | **Sample Site** | **Shannon (1)** | **Shannon (2)** | **chao1** | **Obs._species** | **% of Coverage** | **Nº of sequences after quality control** |
| --- | --- | --- | --- | --- | --- | --- | --- |
| **CR.110** | Tunis | 8,544712 | 3,055406 | 954,358 | 450 | 47,15207 | 1201 |
| **CR.110*** | Tunis | 8,701324 |  | 1960,615 | 901 | 45,95496 | 2693 |
| **CR.154** | Tunis | 5,841641 | 2,562743 | 191,571 | 141 | 73,60179 | 641 |
| **CR.160** | Tunis | 6,2841 | 2,430109 | 179,400 | 91 | 50,72464 | 301 |
| **CR.143** | Villefranche | 2,122316 | 0,975328 | 135,555 | 83 | 61,22951 | 1778 |
| **CR.169** | Villefranche | 6,128629 | 2,868360 | 442,840 | 261 | 58,93764 | 1015 |
| **CR.171** | Villefranche | 5,356268 | 2,702977 | 457,211 | 304 | 66,49005 | 2013 |
| **CR.172** | Villefranche | 7,854999 | 3,311013 | 808,512 | 453 | 56,0288 | 1411 |
| **CR.174** | Villefranche | 7,333845 | 3,138014 | 534,015 | 314 | 58,79975 | 1037 |
| **CR.175** | Villefranche | 5,854284 | 2,878004 | 322,679 | 233 | 72,20793 | 1863 |
| **CR.176** | Villefranche | 5,767355 | 2,685464 | 551,509 | 326 | 59,11054 | 2722 |
| **CR.181** | Liguria | 5,322145 | 2,509050 | 420,386 | 274 | 65,17814 | 2576 |
| **CR.183** | Liguria | 7,247072 | 2,714270 | 460,175 | 337 | 73,23294 | 2454 |
| **CR.183*** | Liguria | 9,871772 |  | 3145,648 | 1806 | 57,41265 | 6467 |
| **CR.186** | Agios Pavlo | 5,59877 | 2,126183 | 505,875 | 323 | 63,84977 | 4988 |
| **CR.187** | Agios Pavlo | 7,592145 | 3,156398 | 275,600 | 148 | 53,70102 | 355 |
| **CR.186*** | Agios Pavlo | 9,535506 |  | 2620,091 | 1552 | 59,23457 | 5637 |
| **CR.199** | Es Cargol | 7,023518 | 2,723778 | 464,333 | 325 | 69,99282 | 2890 |
| **CR.200** | Es Cargol | 6,487306 | 2,508353 | 395,483 | 239 | 60,4323 | 3255 |
| **CR.201** | Es Cargol | 6,985461 | 2,660719 | 336,120 | 243 | 72,29561 | 1730 |
| **CR.200*** | Es Cargol | 9,084988 |  | 2509,327 | 1525 | 60,77325 | 8999 |
| **CR.205** | Es Cargol | 6,531811 | 2,607465 | 565,716 | 366 | 64,69666 | 4702 |
| **CR.201*** | Es Cargol | 6,638923 |  | 771,887 | 378 | 48,97088 | 756 |
| **CR.215** | Illetas | 6,053569 | 2,252359 | 275,250 | 189 | 68,66485 | 1492 |
| **CR.216** | Illetas | 7,744204 | 2,278550 | 557,977 | 304 | 54,48251 | 1505 |
| **CR.217** | Illetas | 7,112268 | 2,567683 | 455,500 | 286 | 62,78814 | 1695 |
| **CR.216*** | Illetas | 8,517622 |  | 2104,369 | 1191 | 56,59651 | 3696 |
| **CR.223** | Marseille | 5,265586 | 1,270739 | 740,924 | 447 | 60,33007 | 4619 |
| **CR.224** | Marseille | 4,210444 | 1,125227 | 458,018 | 293 | 63,97121 | 3732 |
| **CR.225** | Marseille | 6,359365 | 2,255547 | 539,000 | 365 | 67,718 | 3967 |
| **CR.225*** | Marseille | 4,015745 |  | 577,153 | 279 | 48,34066 | 3211 |
| **CR.227** | Marseille | 5,054697 | 1,246744 | 467,651 | 251 | 53,67248 | 2043 |
| **CR.228** | Marseille | 4,312886 | 1,066465 | 311,028 | 178 | 57,22947 | 1618 |
| **CR.227*** | Marseille | 9,314627 |  | 3141,135 | 2014 | 64,11693 | 9383 |
| **CR.231** | Malta | 7,639818 | 2,853211 | 1400,126 | 824 | 58,85181 | 4011 |
| **CR.232** | Malta | 6,441548 | 2,322919 | 1030,315 | 602 | 58,42869 | 3081 |
| **CR.233** | Malta | 6,412122 | 1,729170 | 848,962 | 446 | 52,53468 | 2263 |
| **CR.234** | Malta | 6,508274 | 1,818450 | 1024,536 | 531 | 51,82831 | 3400 |
| **CR.231*** | Malta | 5,832152 |  | 1970,640 | 1234 | 62,61924 | 13487 |
| **CR.237** | Albany | 7,427073 | 2,381933 | 465,018 | 277 | 59,56752 | 1029 |
| **CR.238** | Albany | 6,936284 | 2,712543 | 630,318 | 416 | 65,99836 | 1683 |
| **CR.238*** | Albany | 8,366488 |  | 3084,255 | 1749 | 56,70736 | 9111 |
| **CR.242** | Rottnest Island2 | 7,111004 | 2,532744 | 425,775 | 290 | 68,11095 | 1675 |
| **CR.242*** | Rottnest Island2 | 8,321256 |  | 3505,467 | 1939 | 55,31359 | 11354 |
| **CR.245** | Cottesloe Beach | 7,507983 | 2,579606 | 932,684 | 496 | 53,1798 | 2534 |
| **CR.246** | Cottesloe Beach | 7,963099 | 2,701464 | 910,97872340 | 516 | 56,64238 | 1890 |
| **CR.245*** | Cottesloe Beach | 7,052192 |  | 948,525 | 586 | 61,7801 | 3498 |
| **CR.248** | Rottnest Island1 | 6,118822 | 2,014646 | 527,428 | 269 | 51,00217 | 2835 |
| **CR.249** | Rottnest Island1 | 5,954218 | 2,377835 | 554,000 | 305 | 55,05415 | 2061 |
| **CR.250** | Rottnest Island1 | 7,053585 | 2,286658 | 355,487 | 226 | 63,57473 | 1220 |
| **CR.249*** | Rottnest Island1 | 7,630212 |  | 3041,403 | 1603 | 52,70593 | 9935 |
| **SD.119** | Villefranche | 6,298756 |  | 3098,487 | 1178 | 38,01854 | 6268 |
| **SD.140** | Tunis | 10,19931 |  | 3455,283 | 1384 | 40,0546 | 3238 |
| **SD.279** | Malta | 9,677451 |  | 3237,044 | 1463 | 45,19555 | 4082 |
| **SD.280** | Cottesloe Beach | 9,583268 |  | 3856,820 | 2277 | 59,03827 | 12440 |

^*^Non-disinfected samples of *C. taxifolia* used as control; SD- sediment; **(1)** - Shannon index calculated with all OTUs hits, **(2)** - Shannon index calculated with main OTU classes found common to disinfected samples.
