# Supplementary material for: Invasion Is a Community Affair: Clandestine Followers in the Bacterial Community Associated to Green Algae, Caulerpa racemosa, Track the Invasion Source
Source: PLoS One. 2013 Jul 16;8(7):e68429. doi: 10.1371/journal.pone.0068429 (PMC3713043; doi:10.1371/journal.pone.0068429)
Supplement: Table S3 — Analysis made with 9999 permutations. (DOCX) [file pone.0068429.s006.docx]

**Table S3-** Statistical results of One-way ANOSIM with Bray-Curtis distance measures applied to each group of treatments’ replicates, using OTU hits. Analysis made with 9999 permutations.

| ***p* values** | Disinfected | Non-disinfected | Sediment | **R** |
| --- | --- | --- | --- | --- |
| Disinfected |  | 0.0001 | 0.0001 | 0.5937 |
| Non-disinfected | 0.0001 |  | 0.0265 |  |
| Sediment | 0.0001 | 0.0265 |  |  |

**H_0_:** that there are no differences between the distances of members of the various groups, not rejected H_0_ if: **p>0.05**. The most close to 1 **R** value is the most similar samples are within the same groups
