# Supplementary material for: Invasion Is a Community Affair: Clandestine Followers in the Bacterial Community Associated to Green Algae, Caulerpa racemosa, Track the Invasion Source
Source: PLoS One. 2013 Jul 16;8(7):e68429. doi: 10.1371/journal.pone.0068429 (PMC3713043; doi:10.1371/journal.pone.0068429)
Supplement: Table S4 — Analysis made with 9999 permutations. (DOCX) [file pone.0068429.s007.docx]

**Table S4-** Statistical results of One-way ANOSIM with Bray-Curtis distance measures applied to each group of sites replicates (only considering disinfected samples), using OTU hits. Analysis made with 9999 permutations.

| ***p* values** | Tunis | Villefranche | Greece | Mallorca | Marseille | Malta | Australia | **R** |
| --- | --- | --- | --- | --- | --- | --- | --- | --- |
| Tunis |  | **0.0724** | 0.0297 | 0.0081 | 0.0183 | 0.0295 | 0.0057 | 0.8469 |
| Villefranche | **0.0724** |  | 0.0026 | 0.0008 | 0.0008 | 0.0033 | 0.0002 |  |
| Greece | 0.0297 | 0.0026 |  | 0.003 | 0.0087 | 0.0286 | 0.002 |  |
| Mallorca | 0.0081 | 0.0004 | 0.003 |  | 0.0015 | 0.0038 | 0.0002 |  |
| Marseille | 0.0183 | 0.0008 | 0.0087 | 0.0015 |  | **0.1666** | 0.0011 |  |
| Malta | 0.0295 | 0.0033 | 0.0286 | 0.0038 | **0.1666** |  | 0.0018 |  |
| Australia | 0.0057 | 0.0002 | 0.002 | 0.0002 | 0.0011 | 0.0018 |  |  |

**H_0_:** no differences between the distances of members of the various groups, not rejected if: **p>0.05**. Most similar samples have R values closer to 1.
